# Applications of Artificial Intelligence as a Prognostic Tool in the Management of Acute Aortic Syndrome and Aneurysm: A Comprehensive Review

**DOI:** 10.3390/jcm14238420

**Published:** 2025-11-27

**Authors:** Cagri Ayhan, Marina Mekhaeil, Rita Channawi, Alp Eren Ozcan, Elif Akargul, Atakan Deger, Incilay Cayan, Amr Abdalla, Christopher Chan, Ronan Mahon, Dilara Ayhan, William Wijns, Sherif Sultan, Osama Soliman

**Affiliations:** 1 Royal College of Surgeons in Ireland (RCSI), University of Medicine and Health Sciences, D02 YN77 Dublin, Ireland; cagri.ayhan@materprivate.ie; 2 Precision Cardiovascular Medicine & Innovation Institute (PCMI), Cardiovascular Research Institute Dublin (CVRI), Mater Private Network, D07 KWR1 Dublin, Ireland; 3 School of Medicine, University of Galway, H91 TK33 Galway, Irelandamr.g.abdalla@gmail.com (A.A.);; 4 School of Medicine, University of Duzce, 81620 Duzce, Turkey; 5 Department of Aerospace Medicine, Gulhane Faculty of Medicine, Saglik Bilimleri University, 34480 Istanbul, Turkey; 6 School of Medicine, Atilim University, 06830 Ankara, Turkey; atkdgr@gmail.com (A.D.); incilayzcayan@gmail.com (I.C.); 7 University Hospital Limerick, V94 F858 Limerick, Ireland; 8 The Lambe Institute for Translational Medicine and CÚRAM, National University of Ireland Galway, H91 TK33 Galway, Ireland; 9 Western Vascular Institute, Department of Vascular and Endovascular Surgery, University Hospital Galway, University of Galway, H91 TK33 Galway, Ireland; 10 The Euro Heart Foundation, 3071 EG Rotterdam, The Netherlands

**Keywords:** acute aortic syndromes, aortic dissection, intramural hematoma, aortic aneurysm, machine learning, artificial intelligence, risk assessment, prognosis

## Abstract

Acute Aortic Syndromes (AAS) and Thoracic Aortic Aneurysm (TAA) remain among the most fatal cardiovascular emergencies, with mortality rising by the hour if diagnosis and treatment are delayed. Despite advances in imaging and surgical techniques, current clinical decision-making still relies heavily on population-based parameters such as maximum aortic diameter, which fail to capture the biological and biomechanical complexity underlying these conditions. In today’s data-rich era, where vast clinical, imaging, and biomarker datasets are available, artificial intelligence (AI) has emerged as a powerful tool to process this complexity and enable precision risk prediction. To date, AI has been applied across multiple aspects of aortic disease management, with mortality prediction being the most widely investigated. Machine learning (ML) and deep learning (DL) models—particularly ensemble algorithms and biomarker-integrated approaches—have frequently outperformed traditional clinical tools such as EuroSCORE II and GERAADA. These models provide superior discrimination and interpretability, identifying key drivers of adverse outcomes. However, many studies remain limited by small sample sizes, single-center design, and lack of external validation, all of which constrain their generalizability. Despite these challenges, the consistently strong results highlight AI’s growing potential to complement and enhance existing prognostic frameworks. Beyond mortality, AI has expanded the scope of analysis to the structural and biomechanical behavior of the aorta itself. Through integration of imaging, radiomic, and computational modeling data, AI now allows virtual representation of aortic mechanics—enabling prediction of aneurysm growth rate, remodeling after repair, and even rupture risk and location. Such models bridge data-driven learning with mechanistic understanding, creating an opportunity to simulate disease progression in a virtual environment. In addition to mortality and growth-related outcomes, morbidity prediction has become another area of rapid development. AI models have been used to assess a wide range of postoperative complications, including stroke, gastrointestinal bleeding, prolonged hospitalization, reintubation, and paraplegia—showing that predictive applications are limited only by clinical imagination. Among these, acute kidney injury (AKI) has received particular attention, with several robust studies demonstrating high accuracy in early identification of patients at risk for severe renal complications. To translate these promising results into real-world clinical use, future work must focus on large multicenter collaborations, external validation, and adherence to transparent reporting standards such as TRIPOD-AI. Integration of explainable AI frameworks and dynamic, patient-specific modeling—potentially through the development of digital twins—will be essential for achieving real-time clinical applicability. Ultimately, AI holds the potential not only to refine risk prediction but to fundamentally transform how we understand, monitor, and manage patients with AAS and TAA.

## 1. Introduction

Acute Aortic Syndrome (AAS) and Thoracic Aortic Aneurysm (TAA) are some of the most life-threatening pathologies that occur in cardiovascular medicine. AAS is an umbrella term for three conditions: acute aortic dissection, intramural hematoma, and penetrating atherosclerotic ulcers. TAA refers to dilation of the thoracic aorta due to intrinsic weakness of the vessel wall, potentially leading to dissection or rupture. The vast majority of AAS are acute aortic dissections caused by tears in the intimal layer of the aorta [1]. Population-based studies have shown an incidence of 2.6–3.5 cases per 100,000 people per year [2]. Although less common than other cardiovascular conditions, timely recognition is essential, as untreated AAS carries a mortality of ~1% per hour, rising to 50% by day three and up to 80% by week two [3]. Mortality risk for these conditions directly correlates with delays in diagnosis and treatment, making prompt recognition and intervention crucial for a favorable prognosis and patient survival. In recent years, AI-based triage models using computed tomography angiography (CTA) data have shown promising potential in identifying acute aortic dissections and related symptoms at presentation, supporting clinicians in making rapid, life-saving decisions.

Challenges in managing AAS and TAA extend beyond early diagnosis; proper monitoring and management of patients in both the pre- and post-operative period is just as important. Current clinical guidelines and protocols rely heavily on anatomical parameters; for example, TAA, a precursor condition, is currently assessed primarily by maximum aortic diameter. Current international guidelines [4,5] recommend operative repair at ≥5.5 cm for sporadic ascending TAA. In special circumstances—such as heritable syndromes, bicuspid aortic valve, rapid aneurysm growth, or smaller body size—lower thresholds are considered, as dissection can occur at smaller diameters [5]. Importantly, many dissections still arise below the 5.5 cm cut-off, underscoring the limitations of diameter-based risk stratification alone [6]. The risk associated with AAS can depend on a multitude of factors, such as hemodynamic stress, tissue composition, and inflammatory remodeling; these parameters would be difficult to distill into a streamlined criterion. Treatment, both surgical and medical, carries in itself its own risks, and maximum therapeutic benefit can depend on numerous complex patient variables. Current clinical risk scores (e.g., IRAD, Penn classification, EuroSCOREII) fail to entirely encompass each individual patient profile. This highlights the need for more accurate and individualized risk assessment. Artificial intelligence (AI), particularly machine learning (ML), has emerged as a promising approach to address this challenge. Machine learning is a subset of artificial intelligence that essentially ‘teaches’ computers how to complete certain tasks independently [7]. Computer models gradually acquire knowledge using algorithms to assist in decision-making [8]. Over time, these models use what they have learned to continuously enhance their accuracy.

The idea that machines could ‘think’ dates back to Alan Turing’s seminal proposal in 1950, but only in the past decade has this concept been applied to a practical framework known as AI. In the rapidly evolving field of medicine, where numerous advancements are constantly being made, machine learning has gradually risen to establish its presence. In recent years, machine learning has been applied to assist in the clinical risk prediction and diagnosis of cardiovascular diseases such as coronary vascular disease, heart failure, coronary heart disease, and cardiac arrhythmias [9]. As of June 2019, the American Food and Drug Administration has approved several AI algorithms for medical use, with seven specifically tailored to cardiology, mainly in the detection of arrhythmias such as AliveCor and atrial fibrillation [10]. These AI-driven methods have been proven to be credible in numerous aspects. With machine learning, we are now able to analyze a vast volume of data that includes advanced imaging tools, diagnostic applications, electronic health records, biobanks, and molecular profiling [11]. Recent studies have shown that AI has been integral to the evolution of cardiovascular risk assessment and outcome prediction. AI has been shown the ability to identify specific biomarkers related to cardiovascular risk, integrate multi-layered data to distinguish patient subpopulations based on unique molecular signatures, and even suggest individual responses to specific treatments. Khera et al. [12] It is very likely that AI/ML will change the practice of medicine, making it faster and more precise, giving physicians better information to personalize patient treatment [13].

**Figure 1:**
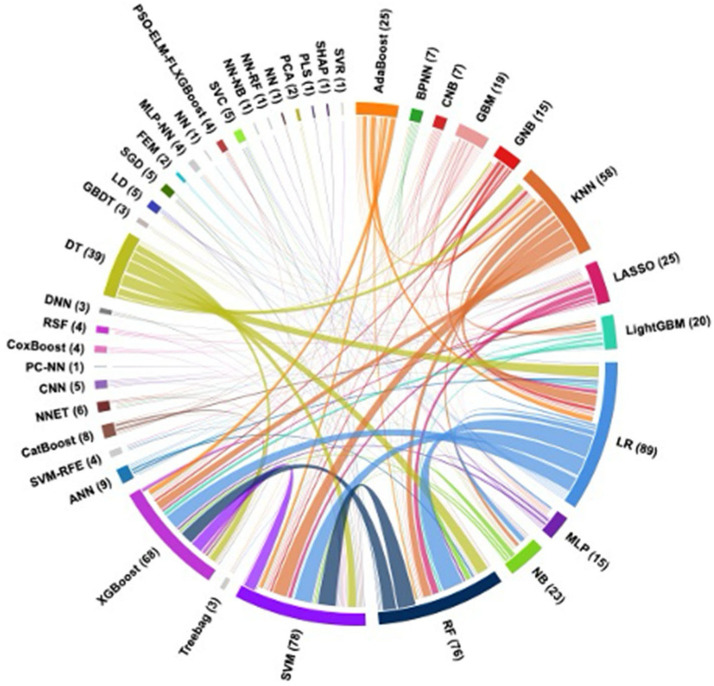
Chord diagram illustrating pairwise comparisons among AI models across all included studies. This chord diagram visualizes how frequently different artificial intelligence (AI) models were compared within the analyzed literature. Each colored connection represents a comparison between two models, with the thickness of the chord corresponding to the frequency of comparison. Numbers in brackets next to each model indicate the total number of times that model was compared with others. The colors are used for visual differentiation only and do not reflect the performance or outcomes of the comparison.

Artificial intelligence (AI) has been applied across a wide spectrum of research in aortic syndromes, with its potential limited only by data availability and human creativity. Consequently, the literature encompasses diverse analytical approaches addressing different clinical outcomes and disease aspects. This comprehensive review is based on a systematic literature search that identified 52 relevant studies. Since it would be impractical to discuss each study in detail, all included papers are summarized in Table 1 and Table 2. Additionally, Figure 1 presents a chord diagram illustrating how frequently different AI models were compared against each other across the literature. In this review, we focus on the most frequent and clinically relevant AI applications, including the prediction of mortality, composite endpoints, acute kidney injury and renal failure, aneurysm growth and remodeling, and rupture risk. Our goal is to provide an integrated overview of the current state of AI in this field, highlighting the unique contributions and limitations of each approach, and to outline future directions for advancing AI-based prognostication in aortic diseases.

**Table 1:** Study Characteristics and Data Overview.

Author	Year	Short Purpose	Disease	Study Type	Dataset Origin (Single-Center vs. Multi-Centre)	Dataset Size
Tan et al. [14]	2021	Mortality Prediction	Stanford Type A Aortic Dissection	Retrospective cohort study	Single	206
Macrina et al. [15]	2009	Mortality Prediction	Stanford Type A Aortic Dissection	Retrospective cohort study	Multi	208
Naazie et al. [16]	2022	Mortality Prediction	Descending Thoracic Aortic Aneurysm	Retrospective cohort study	Multi	2141
Wang et al. [17]	2022	Mortality Prediction	Acute Aortic Syndrome	Retrospective cohort study	Multi	1298
Yang et al. [18]	2023	Mortality Prediction	Stanford Type B Aortic Dissection	Retrospective cohort study	Single	978
Guo et al. [19]	2921	Mortality Prediction	Acute Aortic Dissection	Retrospective cohort study	Single	1344
Liu et al. [20]	2022	Mortality Prediction	Stanford Type B Aortic Dissection	Retrospective cohort study	Single	428
Macrina et al. [21]	2010	Mortality Prediction	Stanford Type A Ascending Aortic Dissection	Retrospective cohort study	Multi	235
Liu et al. [22]	2022	Mortality Prediction	Stanford Type A Aortic Dissection	Prospective cohort study	Multi	5014
Wu et al. [23]	2023	Mortality Prediction	Acute Aortic Dissection	Retrospective cohort study	Single	380
Lei et al. [24]	2024	Mortality Prediction	Acute Aortic Dissection	Retrospective cohort study	Multi	643
Chen et al. [25]	2025	Mortality Prediction	Stanford Type A Aortic Dissection	Retrospective cohort study	Single	925
Cai et al. [26]	2025	Mortality Prediction	Stanford Type A Aortic Dissection	Retrospective cohort study	Multi	274
Zhang et al. [27]	2025	Mortality Prediction	Stanford Type A Aortic Dissection	Retrospective cohort study	Single	289
Zhang et al. [28]	2025	Mortality Prediction	Stanford Type A Aortic Dissection	Retrospective cohort study	Single	640
Liu et al. [29]	2024	Mortality Prediction	Stanford Type A Aortic Dissection	Retrospective cohort study	Multi	3310
Koru et al. [30]	2024	Rupture Risk Prediction	Thoracic Aortic Aneurysm	Simulation study	N/A	3
He et al. [31]	2021	Rupture Risk and location Prediction	Ascending Thoracic Aortic Aneurysm	In vitro study using patient specimens	Single	15
Liang et al. [32]	2017	Rupture Risk Prediction	Ascending Aortic Aneurysm	Simulation study	Single	25
Wu et al. [33]	2019	Rupture Risk Prediction	Stanford Type A Aortic Dissection	Retrospective cohort study	Single	1133
Dong et al. [34]	2023	Rupture Risk Prediction	Stanford Type A Aortic Dissection	Retrospective cohort study	Single	564
Lin et al. [35]	2023	Rupture Risk Prediction	Stanford Type A Aortic Dissection	Retrospective cohort study	Single	200
O’Rourke et al. [36]	2022	Rupture Location Prediction	Ascending Aortic Aneurysm	Retrospective cohort study	Single	12
Chiu et al. [37]	2021	Growth Rate Prediction	Thoracic Aortic Aneurysm	Ex vivo mechanical study	N/A	31
Geronzi et al. [38]	2023	Growth Rate Prediction	Ascending Aortic Aneurysm	Retrospective cohort study	Multi	50
Geronzi et al. [39]	2023	Growth Rate Prediction	Ascending Aortic Aneurysm	Retrospective cohort study	Multi	70
Li et al. [40]	2022	Post-operation Acute Renal Failure Prediction	Acute Aortic Syndrome	Retrospective cohort study	Multi	1637
Zhou et al. [41]	2019	Post-operation Acute Renal Failure and Paraplegia Prediction	Thoracoabdominal Aortic Aneurysm	Retrospective cohort study	Single	212
Xinsai et al. [42]	2022	Post-operation Acute Kidney Injury Prediction	Stanford Type A and Stanford Type B Acute Aortic Dissection	Retrospective cohort study	Single	456
Wei et al. [43]	2025	Acute Kidney Injury Prediction	Acute Aortic Dissection	Retrospective cohort study	Multi	325
Chen et al. [44]	2025	Acute Kidney Injury Prediction	Stanford Type A Aortic Dissection	Retrospective cohort study	Single	1350
Li et al. [45]	2025	Continuous Renal Replacement Therapy Prediction	Stanford Type A Aortic Dissection	Retrospective cohort study	Single	588
Liu et al. [46]	2024	Acute Kidney Injury Prediction	Stanford Type A Aortic Dissection	Retrospective cohort study	Single	572
Zhou et al. [47]	2023	Negative Distal Aortic Remodeling and Reintervention Prediction	Stanford Type B Aortic Dissection	Retrospective cohort study	Single	147
Zhou et al. [48]	2021	Distal Aortic Enlargement Prediction	Stanford Type B Aortic Dissection	Retrospective cohort study	Single	503
Gao et al. [49]	2025	Prediction of Intramural Hematoma	Acute Aortic Intramural Hematoma	Retrospective cohort study	Single	119
Chen et al. [50]	2025	Prediction of Negative Remodeling in Intramural Hematoma	Stanford Type B Aortic Dissection	Retrospective cohort study	Single	154
Dong et al. [51]	2021	Reintervention Prediction	Stanford Type B Aortic Dissection	Retrospective cohort study	Single	192
Wen et al. [52]	2025	Prediction of postop reintubation	Stanford Type A Aortic Dissection	Retrospective cohort study	Multi	861
Zhao et al. [53]	2021	Acute Ischemic Stroke Prediction	Stanford Type A Aortic Dissection	Retrospective cohort study	Single	300
Chen et al. [54]	2021	Intensive Care Unit (ICU) Stay Length Prediction	Stanford Type A Aortic Dissection	Retrospective cohort study	Single	353
Li et al. [55]	2025	Hospital Stay Prediction	Acute Aortic Dissection	Retrospective cohort study	Single	516
Schäfer et al. [56]	2023	Prediction of multiple events	Stanford Type A Aortic Dissection and Aortic Arch Aneurysm	Retrospective cohort study	Single	93
Ding et al. [57]	2023	Prognosis of Aortic Intramural Hematoma	Aortic Intramural Hematoma	Retrospective cohort study	Single	120
Xie et al. [58]	2024	Prediction of multiple events	Stanford Type A Aortic Dissection	Retrospective cohort study	Single	380
Carroll et al. [59]	2025	Prediction of multiple events	Hemiarch surgery (Postoperative outcomes)	Retrospective cohort study	Single	602
Lu et al. [60]	2024	Prediction of multiple events	Stanford Type B Aortic Dissection	Retrospective cohort study	Multi	369
Luo et al. [61]	2025	Prediction of multiple events	Stanford Type A Aortic Dissection	Retrospective cohort study	Single	635
Li et al. [62]	2025	Prediction of postoperative gastrointestinal bleeding	Stanford Type A Aortic Dissection	Retrospective cohort study	Single	525
Liu et al. [63]	2024	Acute Lung Injury Prediction	Stanford Type A Aortic Dissection	Retrospective cohort study	Multi	2499
Jin et al. [64]	2025	Prediction of Mesenteric Malperfusion	Acute Aortic Dissection	Retrospective cohort study	Single	435
Jin et al. [65]	2025	Prediction of Mesenteric Malperfusion	Acute Aortic Dissection	Retrospective cohort study	Multi	525

The table outlines the main characteristics of the included studies. The purpose, along with the year and disease focus, is presented to highlight the evolving trends and research aims over time. In addition, we included the methodological approach, dataset size, and dataset type (single- vs. multi-center), as these represent key pillars for evaluating AI-based studies before delving into their technical details.

**Table 2:** Algorithm Comparison and Key Findings.

	List of AI Algorithms Compared	Best AI Model	Type of Data Used in Best AI Model	Findings
Tan et al. [14]	LightGBM, XGBoost, CatBoost	Fusion of LightGBM, XGBoost, and CatBoost	RBC transfusion, cardiopulmonary bypass time, rectal temperature, plasma transfusion, cross-clamping time, waiting time before operation, nasopharyngeal temperature, coronary ostial nari type, age, blood pericardial effusion.	Fusion model revealed an accuracy of 1 on predicting post-operative early mortality.
Macrina et al. [15]	Neural Network Logistic Regression	Neural Network	Presence of pre-operative shock Intubation status. Neurological symptoms. Immediate post-operative presence of dialysis in continuous mode. Quantity of post-operative bleeding in the first 24 h. Length of extracorporeal circulation. Post-operative chronic renal failure. Year of surgery.	NN model showed an AUC of 0.905 in predicting long-term mortality.
Naazie et al. [16]	Logistic Regression	Logistic Regression (LR)	Age ≥ 75, coronary artery disease, ASA class, urgency of procedure, prior carotid revascularization, proximal landing zone.	LR model revealed an AUC of 0.79 in predicting 30-day mortality after TEVAR.
Wang et al. [17]	Logistic Regression	Logistic Regression	Digestive system symptoms, pulse deficit, creatinine levels, lesion extension to iliac vessels, pericardial effusion, Stanford type A.	LR model revealed and AUC of 0.821 in external validation to predict early in hospital mortality.
Yang et al. [18]	LASSO Regression, Logistic Regression	Logistic Regression	Heart rate >100 bpm, systolic blood pressure ≥160 mmHg, pleural effusion, anemia, abnormal cTnT, eGFR <60 mL/min.	LR model showed an AUC of 0.894 in predicting early in hospital mortality.
Guo et al. [19]	XGBoost, Logistic Regression, Decision Tree, Gaussian Naive Bayes, KNN	XGBoost	Treatment strategy, Type of AAD, Ischemia-modified albumin levels.	XGBoost yielded an AUC of 0.927, sensitivity of 0.966 and specificity of 0.855 in predicting in hospital mortality.
Liu et al. [20]	U-Net CNN for segmentation	U-Net CNN	Lean Psoas Muscle Area (LPMA), Psoas Muscle Density (PMD), BMI, Psoas Muscle Index (PMI).	Patients who have lower LPMA were shown to have 5.62 times higher mortality risk.
Macrina et al. [21]	Neural Networks, SVM	Neural Network for better AUC, SVM for better Gini’s coefficient	Immediate post-op chronic renal failure, circulatory arrest time, type of surgery on ascending aorta plus hemi-arch, extracorporeal circulation time, Marfan habitus.	NN model yielded an AUC of 0.870 in predicting 30-day mortality following surgery for AD.
Liu et al. [22]	XGBoost, LASSO Regression, SVM, KNN, AdaBoost	XGBoost	Systemic thrombo-inflammatory (STI) index, creatinine level, hemoglobin, cerebral and coronary perfusion, shock, aortic regurgitation.	XGBoost achieved an AUC of 0.873 for predicting operative mortality, outperforming other models.
Wu et al. [23]	XGBoost, Random Forest, Logistic Regression, Decision Tree, SVM	XGBoost	Stanford type A, maximum aortic diameter >5.5 cm, heart rate variability, diastolic blood pressure variability, involvement of the aortic arch.	XGBoost exhibited an AUC of 0.926 in predicting in hospital mortality.
Lei et al. [24]	Simple decision tree, Random Forest, XGBoost, and Multivariable logistic regression	Random Forest	Mean 24 h fluid intake (the most important factor), blood phosphate, initial heart rate (base HR), mean heart rate (average HR), initial systolic blood pressure (base SBP), initial diastolic blood pressure (base DBP), mean systolic blood pressure (average SBP), mean diastolic blood pressure (average DBP), creatinine, alanine aminotransferase (ALT), and aspartate aminotransferase (AST).	RF demonstrated the best predictive performance for in-hospital mortality among ICU patients with aortic dissection, achieving AUCs of 0.870 (internal) and 0.767 (external) validation.
Chen et al. [25]	Simple decision tree, Random Forest, XGBoost, SVM and Multivariable logistic regression	SVM (Fit.SVM)	Cerebral malperfusion, mesenteric malperfusion, critical preoperative status, D-dimer, platelet count, CABG, intraoperative blood product transfusion, and cardiopulmonary bypass time.	SVM-based Fit. SVM model achieved the highest predictive accuracy for in-hospital mortality after extended aortic arch repair in ATAAD patients, with an AUC of 0.782 in the testing cohort.
Cai et al. [26]	SVM	SVM	Operation time, cardiopulmonary bypass duration, aortic cross-clamp time, age, plasma transfusion volume, serum creatinine, and white blood cell count.	The SVM model achieved the highest accuracy for predicting long-term survival after surgical repair of Type A aortic dissection, with AUCs of 0.853 (internal) and 0.877 (external) validation.
Zhang et al. [27]	Tree-based Bagging GBM, Adaboost, Logistic Regression	Tree-based Bagging	Age, white blood cell count, systolic blood pressure, lymphocytes, carbon dioxide combining power, eosinophils, β-blocker use, and surgical therapy.	The Treebag model achieved the highest accuracy for predicting 1-year mortality in Type A aortic dissection, with an AUC of 0.91 in the validation cohort.
Zhang et al. [28]	XGBoost, Logistic Regression, SVM, Random Forest, improved PSO-ELM-FL XGBoost model	PSO-ELM-FLXGBoost (enhanced XGBoost model)	Gender, age, body mass index, lower limb ischemia, eGFR < 50 mL/min/1.73 m^2^, alanine aminotransferase, lactate dehydrogenase, D-dimer, red blood cell transfusion, and cardiopulmonary bypass time.	The optimized PSO-ELM-FLXGBoost model achieved the highest predictive accuracy for 30-day postoperative mortality after total aortic arch replacement with frozen elephant trunk implantation, with an AUC of 0.869 in the validation cohort.
Liu et al. [29]	Logistic Regression, XGBoost	XGBoost	Platelet–leukocyte ratio (STI index), D-dimer, activated partial thromboplastin time, urea nitrogen, glucose, lactate, base excess, hemoglobin, albumin, and creatine kinase-MB.	The Bio-XGBoost model demonstrated excellent discrimination for operative mortality after surgical repair of acute Type A aortic dissection, achieving an AUC of 0.884 in the validation cohort. personalized postoperative management.
Koru et al. [30]	ANN	ANN	Ascending aortic diameter, wall shear stress, von Mises stress, deformation.	Ascending aorta diameter were shown to be correlated with rupture risk.
He et al. [31]	LASSO Regression, Random Forest, MLP	LASSO Regression	Tension, strain, slope, and curvature at two points in the low-strain region.	LR model revealed the most optimum results in terms of predicting pressure risk ratio (PRR).
Liang et al. [32]	SVM, SVR	SVM	Statistical Shape Model parameters, maximum diameter, centerline curvature, surface curvature.	SVM showed an accuracy of 95.58% to classify patients as high or low rupture risk. SVR revealed a regression error of 0.0332 to estimate precise rupture risk.
Wu et al. [33]	Random Forest, LASSO	Random Forest	Periaortic hematoma, aortic height index (AHI), syncope, pleural effusion.	RF could predict in hospital rupture risk with an AUC of 0.752, sensitivity of 0.99 and specificity of 0.514 in testing.
Dong et al. [34]	LASSO Regression, Logistic Regression	Logistic Regression	Ascending aorta diameter, false lumen diameter, false lumen/true lumen diameter ratio, number of branch arteries involved.	LR could identify independent risk factors for pre-operative rupture based on CTA imaging features.
Lin et al. [35]	Logistic Regression, Random Forest, SVM, CNN	CNN	Age, sex, lactates > 1.9 mmol/L, aortic diameter, ventilator-assisted ventilation, WBC > 14.2 × 10^9^/L.	CNN model showed the best performances according to AUC (0.99) to predict rupture risk.
O’Rourke et al. [36]	Finite Element Modeling	Finite Element Modeling (FEM)	Pre- and post-rupture CT data, 3D aortic geometry, strain distribution.	Rupture location of patients who has sorter follow up interval (<3 years) could be predicted by FEM.
Chiu et al. [37]	LASSO	LASSO	Aortic relative strain, elastic modulus, fracture toughness, anatomical location.	Increased relative strain was found to be a significant risk factor aneurysm growth.
Geronzi et al. [38]	Decision Tree, Linear Discriminant, Logistic Regression, Naive Bayes, SVM, KNN	Support Vector Machine (SVM)	Diameter, Diameter/Centerline Ratio (DCR), External-Internal Line Ratio (EILR), Tortuosity (T).	SVM could differentiate patients into two categories as high growth risk vs. low growth risk with an AUC of 0.94.
Geronzi et al. [39]	SVM (Gaussian), PLS Regression	PLS Regression	Maximum diameter, diameter/centerline ratio, external/internal curvature line ratio, tortuosity.	PLS regression could precisely predict growth rate with an mean square error of 0.066 mm/month.
Li et al. [40]	XGBoost, Logistic Regression, AdaBoost, Random Forest	XGBoost	Age, emergency surgery, cardiopulmonary bypass time, leukocyte count, platelet count, estimated glomerular filtration rate (eGFR).	XGBoost could predict occurrence of acute renal failure with an AUC of 0.82, sensitivity of 82.9% and specificity of 67.6%.
Zhou et al. [41]	Logistic Regression, Linear SVM, Gaussian SVM, Random Forest	Random Forest (for Acute Renal Failure prediction), Linear SVM (for paraplegia prediction)	Lactic acid (LAC) after surgery, BMI, RBC transfusion, operation time, age, Marfan syndrome, platelets.	RF showed an AUC of 0.89 to predict acute renal failure and SVM yielded an AUC of 89 to predict paraplegia after surgery.
Xinsai et al. [42]	Random Forest, LightGBM, Decision Tree, XGBoost	Random Forest (Stanford Type A Aortic Dissection), LightGBM (Stanford Type B Aortic Dissection)	Baseline Serum Creatinine (SCR), Blood Urea Nitrogen (BUN), Uric Acid (UA), Mechanical Ventilation Time (MVT).	RF showed best prediction performance for acute kidney injury (AKI) in Stanford Type A Aortic Dissection group with an AUC of 0.76 LightGBM showed best prediction performance for acute kidney injury (AKI) in Stanford Type B Aortic Dissection group with an AUC of 0.734.
Wei et al. [43]	SVM, GBM, Neural Network XGBoost, KNN, LightGBM), and CatBoost	CatBoost	Weight, BMI, APSIII, minimum BUN, maximum BUN, minimum creatinine, maximum creatinine, maximum glucose, urine output.	The CatBoost model achieved the best performance for predicting in-hospital acute kidney injury, with AUCs of 0.723 (internal) and 0.712 (external) validation.
Chen et al. [44]	Gradient Boosting Machine (GBM), LightGBM, Random Forest (RF), K-Nearest Neighbors (KNN), Multi-Layer Perceptron Neural Network (MLP-NN), Naive Bayes (NB), and Logistic Regression (LR)	LightGBM	Ventilation time, minimum hourly urine output (first 48 h), diuretic use, serum creatinine, heart rate, serum urea, administration of rhBNP and urapidil, mean corpuscular hemoglobin concentration, and blood glucose.	The LightGBM model achieved the highest accuracy for predicting acute kidney injury following ATAAD surgery, with an AUC of 0.886 in the validation cohort.
Li et al. [45]	XGBoost, Not specified (Evaluated seven ML methods) [220, Previous knowledge].	XGBoost model [Previous knowledge]	Peak intraoperative lactate, red blood cell transfusion volume, renal artery involvement, myoglobin, cystatin C, and creatine kinase-MB.	The XGBoost model achieved the highest predictive accuracy for postoperative continuous renal replacement therapy after ATAAD surgery, with an AUC of 0.96 in the validation cohort.
Liu et al. [46]	Artificial Neural Network (ANN), Logistic Regression, Lasso regression, Support Vector Machine-Recursive Feature Elimination (SVM-RFE), Random Forest (RF) [Previous knowledge]	Artificial Neural Network (ANN) [Previous knowledge]	Baseline serum creatinine, and ICU admission variables including serum cystatin C, procalcitonin, aspartate transaminase, platelet count, lactate dehydrogenase, urine N-acetyl-β-D-glucosidase, and APACHE II score.	The ANN model demonstrated the best predictive accuracy for severe acute kidney injury (stage III) after total aortic arch replacement in ATAAD, achieving an AUC of 0.916 in the validation cohort.
Zhou et al. [47]	CNN, PC-NN	PC-NN	Aortic boundary point clouds, clinical features.	PC-NN outperformed CNN both in predicting negative remodeling and reintervention indication with AUCs of 0.876 and 0.8, respectively.
Zhou et al. [48]	Logistic Regression (LR), Artificial Neural Network (ANN), Random Forest (RF), Support Vector Machine (SVM)	Logistic Regression (LR) for distal aortic enlargement, ANN for aneurysm formation	True lumen collapse, multi-false lumens, persistent false lumen perfusion, primary entry tear location.	LR revealed an AUC of 0.773 and sensitivity of 96.7%, specificity of 49.2% in predicting distal aortic enlargement. ANN yielded an AUC of 0.876, sensitivity of 90.5% and specificity of 79.1%.
Gao et al. [49]	Random Forest (RF), Support Vector Machine (SVM), Logistic Regression (LR), K-nearest neighbor (KNN), Decision Tree (DT), and Stochastic Gradient Descent (SGD)	Combined model (Radiomics + Clinical + CTA) via KNN (in test set)	Chest distress, white blood cell (WBC) count, low-density lipoprotein (LDL), associated dissection, penetrating aortic ulcer (PAU) location, Wavelet_glszm_wavelet HLL–SmallAreaEmphasis, Original_glrlm_GrayLevelNonUniformityNormalized, Original_shape_LeastAxisLength, Laplaciansharpening_glszm_GrayLevelNonUniformity, and Wavelet_glszm_wavelet HLL–SmallAreaHighGrayLevelEmphasis.	The integrated radiomics + clinical + CTA model achieved superior predictive accuracy for IMH progression, with an AUC of 0.917 in the validation cohort.
Chen et al. [50]	Univariate logistic regression, K-nearest neighbors (KNN), support vector machine (SVM), random forest (RF), decision tree (DT), gradient boosting machine (GBM), XGBoost, CatBoost, and the multilayer perceptron (MLP)	CatBoost model	Monocytes (count on admission), lymphocytes (count on admission), eosinophils (count on admission), white blood cells (count on admission), neutrophil-to-lymphocyte ratio (on admission), hypertension, and statins treatment.	The CatBoost model achieved excellent performance for predicting negative remodeling during the acute phase of uncomplicated Stanford type B intramural hematoma, with an AUC of 0.969 in the validation cohort.
Dong et al. [51]	LASSO Regression (LR), Random Forest (RF), XGBoost, AdaBoost, KNN, Naive Bayes, SVM, BPNN (Back Proagation Neural Networks)	Logistic Regression (LR)	Maximum false lumen diameter, total aortic diameter, number of bare-metal stents, residual perfusion of false lumen.	LR could predict indication for reintervention with an AUC of 0.802.
Wen et al. [52]	Multivariable logistic regression, decision tree, random forest), XGBoost, SVM, KNN, and LightGBM	XGBoost	Re-admission to ICU, continuous renal replacement therapy, ICU length of stay, and duration of invasive mechanical ventilation.	XGBoost achieved excellent predictive accuracy for postoperative reintubation after surgical repair of acute type A aortic dissection, with AUCs of 0.969 (testing) and 0.964 (external validation). SHAP analysis highlighted ICU stay, mechanical ventilation duration, and CRRT as the most influential predictors. A web-based calculator was developed to facilitate rapid clinical risk assessment.
Zhao et al. [53]	Deep Neural Network, SVM, Random Forest, LASSO	Deep Neural Network	True lumen diameter ratio of ascending aorta, common carotid artery dissection, low density of internal carotid artery, age.	DNN could predict acute ischemic stroke with an AUC of 0.964, sensitivity of 96% and specificity of 90.6% in validation dataset.
Chen et al. [54]	Random Forest, Naive Bayes, Linear Regression, Decision Tree, Gradient Boosting Decision Tree	Random Forest	D-dimer, serum creatinine, lactate dehydrogenase, cardiopulmonary bypass time, fasting blood glucose, surgical time.	ICU length of stay(LOS) was divided into four categories <4, 4–7, 7–10, and >10 days) RF could predict ICU LOS with an AUC of 0.837 in validation sets.
Li et al. [55]	XGBoost, AdaBoost, KNN, logistic regression, LightGBM, gaussian naive Bayes, MLP, complement naive Bayes, and SVM	XGBoost	Systolic blood pressure, operation time, diameter of the aorta, neutrophil-to-lymphocyte ratio, platelet-to-lymphocyte ratio, HDL-C, albumin, BMI, diabetes mellitus, aortic and tricuspid regurgitation, lymphocyte count, hemoglobin, total cholesterol, glucose, urea, total protein, ALT, prothrombin time, fibrinogen, and INR.	XGBoost showed the highest predictive performance for prolonged hospital stay (>30 days), achieving an AUC of 0.71 with a sensitivity of 0.76 and specificity of 0.84. HDL-C, ALT, systolic blood pressure, lymphocyte percentage, and operation time emerged as the strongest predictors, reflecting their key roles in postoperative recovery dynamics.
Schäfer et al. [56]	PCA	PCA	Aortic centerline angles, arch height-to-length ratio, and aortic tilt.	Multiple aortic events were predicted using 3D aortic shape variations.
Ding et al. [57]	Random Forest, KNN, Gaussian Naive Bayes, Decision Tree, Logistic Regression, SVM (RBF)	SVM (RBF)	Radiomic features from computed tomography angiography (CTA).	SVM model based on radiomics features showed an AUC of 0.787 to predict prognosis of intramural hematoma (IMH).
Xie et al. [58]	XGBoost, Logistic Regression, Random Forest, Gaussian Naive Bayes, SVM, KNN	XGBoost model	Age, left ventricular ejection fraction, acute aortic regurgitation, white blood cell count, creatinine, total operation time, deep hypothermic circulatory arrest time, aortic root procedure, and platelet transfusion volume.	XGBoost showed the best predictive accuracy for postoperative adverse outcomes after emergency total arch repair for acute Type A aortic dissection, achieving an AUC of 0.761. SHAP analysis highlighted acute aortic regurgitation, total operation time, and WBC count as the most influential predictors.
Carroll et al. [59]	Various eXtreme Gradient Boosting (XGBoost) candidate models, Logistic Regression (for comparison)	XGBoost	Demographic, preoperative, and intraoperative parameters including age, gender, comorbidities (HTN, CAD), hemoglobin, creatinine, INR, cardiopulmonary bypass time, aortic cross-clamp time, cerebral protection strategy, adjunctive procedures, and intraoperative blood product transfusion.	XGBoost achieved the best predictive accuracy for life-altering events (stroke, mortality, or new renal replacement therapy) following hemiarch replacement surgery, with an AUC of 0.76 and 88% cross-validation accuracy. SHAP analysis identified nadir hemoglobin, age, and intraoperative red blood cell transfusion as the most influential predictors.
Lu et al. [60]	XGBoost combined with Deep Learning (3D deep CNN U-Net architecture) and LASSO feature selection	Combined model (Rad-Score + Clinical Factors via XGBoost)	Radiomic features from CTA (false lumen volume, largest false lumen diameter at celiac axis, minimal true lumen diameter at celiac axis and distal to renal artery, sphericity of initial flap, and texture-derived GLCM features) combined with clinical parameters (albumin and C-reactive protein).	The integrated model achieved the best performance for predicting postoperative adverse events after TEVAR in acute uncomplicated Type B aortic dissection, with an AUC of 0.985 in external validation.
Luo et al. [61]	Ensemble methods combining 10 algorithms (including Random Survival Forest and, Generalized Boosted Regression, Lasso, SVM, CoxBoost), resulting in 190 combinations	Combination of Random Survival Forest and Generalized Boosted Regression Modeling	Clinical and laboratory features including International Normalized Ratio, creatine kinase-MB, D-dimer, direct bilirubin, hemoglobin, albumin, platelet count, total bilirubin, activated partial thromboplastin time, neutrophil count, and ascending aorta diameter.	The ensemble RSF + GBM model achieved excellent performance for predicting in-hospital major adverse outcomes after TAR + FET in ATAAD, with an AUC of 0.851 in the validation cohort.
Li et al. [62]	Random Forest, SVM, KNN, and Decision Tree	Random Forest model [Previous knowledge]	Mechanical ventilation duration, time to aortic occlusion, red blood cell transfusion, sedative and analgesic drug use, intra-aortic balloon pump, external temporary pacemaker, continuous renal replacement therapy, low cardiac output syndrome, and ICU length of stay.	The Random Forest model achieved excellent predictive accuracy for postoperative gastrointestinal bleeding after Type A aortic dissection surgery, with an AUC of 0.933 in the validation cohort.
Liu et al. [63]	XGBoost	XGBoost model (simplified model)	Clinical and laboratory variables including leukocyte, platelet, hemoglobin, base excess, age, creatinine, glucose, and left ventricular end-diastolic dimension (LVEDD).	The XGBoost model demonstrated high discrimination for predicting acute lung injury after ATAAD surgery, with an AUC of 0.799 in the validation cohort.
Jin et al. [64]	Logistic regression, support vector classification, random forest, XGBoost, naive Bayes, and (MLP)	Random Forest model	Computed Tomography Angiography (CTA) features of the abdominal aorta and bowel, combined with laboratory parameters including white blood cell count, neutrophil count, D-dimer, and lactate levels.	Logistic regression (LR), support vector classification (SVC), random forest (RF), extreme gradient boosting (XGBoost), naive Bayes (NB), and multilayer perceptron (MLP).
Jin et al. [65]	Deep Learning models (MAM model, Integrated model), Logistic Regression (Benchmark clinical model)	Integrated Model (Fusing image features and clinicopathological features)	CTA-derived image features from segmented abdominal aorta (celiac trunk and mesenteric branches) and bowel (jejunum, ileum, and colon), combined with laboratory biomarkers including white blood cell count, neutrophil count, D-dimer, and lactate levels.	Deep Learning models (MAM model, Integrated model), Logistic Regression (Benchmark clinical model) [Previous knowledge].

The table summarizes the list of AI models that were compared within each study. As AI encompasses a wide range of techniques, the performance of a particular model may vary depending on the clinical context and dataset characteristics. Reviewing which models have been compared and identifying those that outperformed others across different studies can provide valuable guidance for future research. In addition, we summarized the datasets used, limiting the list to the top ten variables when applicable, to illustrate the types of data currently utilized and highlight opportunities for incorporating additional information in future studies. Key findings are briefly summarized here, while the most significant studies are discussed in greater detail in the main manuscript. Abbreviations: LightGBM = Light Gradient Boosting Machine; XGBoost = Extreme Gradient Boosting; CatBoost = Categorical Boosting Algorithm; KNN = K-Nearest Neighbors; CNN = Convolutional Neural Network; SVM = Support Vector Machine; GBM = Gradient Boosting Machine; AdaBoost = Adaptive Boosting; PSO-ELM-FL XGBoost = Particle Swarm Optimization-Extreme Learning Machine-Focal Loss XGBoost model; ANN = Artificial Neural Network; MLP = Multilayer Perceptron; SVR = Support Vector Regression; PLS = Partial Least Squares; PCA = Principal Component Analysis; RBF = Radial Basis Function; MAM = Multi-organ feature-based acute aortic dissection complicating mesenteric malperfusion model.

## 2. Overview of Artificial Intelligence and Machine Learning Methodologies in Acute Aortic Syndromes

Artificial intelligence (AI) refers to algorithmic computational systems designed to perform cognitive tasks that usually require human intelligence, including reasoning, problem-solving, and pattern recognition. This term originated in the mid-20th century, and it was used to describe machine-based systems capable of imitating aspects of human thought; however, the modern version of this concept involves an umbrella of algorithmic paradigms [66]. Under this umbrella, machine learning (ML) and deep learning (DL) represent subfields with progressively increasing complexity and autonomy [67]. ML can be considered a subset of AI that enables systems to learn from data and continuously improve performance without explicit programming, while DL, in turn, is a specialized branch of ML that utilizes a multi-layered neural network to automatically extract and combine hierarchical features from more complex and clusters of datasets [66].

When delving deeper into DL-based systems, convolutional neural networks (CNNs) play a particularly key role in medical imaging. This is due to their exceptional ability of grasping spatial hierarchies, which makes them suitable for visual pattern recognition and segmentation tasks [67]. To understand this hierarchy better, this can be reimagined as a layered system: AI encompasses the full breadth of computational intelligence; the main source of AI “learning” would be provided by ML; DL would streamline this mechanism of “learning” and thus allowing for unprecedented levels of abstraction; while CNNs would be optimized DL systems that are task-specific towards spatially structured inputs [66]. This gradual evolution in AI reflects the wider transformative nature of medical innovation towards precision and evidence derived from these more complex and previously difficult to derive datasets [68].

When applying this hierarchy to the topic of this review, early exploratory phases show very promising results. Classical ML has shown unique benefits in cardiovascular medicine; however, applications in acute aortic syndromes are still in an exploratory phase [64]. The methods devised by classic ML could be categorized into three main methods: supervised, unsupervised, and reinforcement learning. By far, the most popular method used in cardiovascular research would be supervised learning [69]. This involves using pre-existing datasets where the inputs and associated end results are known. These algorithms then “learn” the patterns existing between these inputs and known outcomes, which could then be extrapolated onto unseen datasets to generate predictions such as postoperative mortality, aneurysm rupture, or adverse remodeling. On the other hand, unsupervised learning does not require labeled outcomes; instead, it explores the intrinsic nature of these datasets to uncover hidden patterns or clusters [70]. For example, it could help to uncover unknown patient subgroups with unique risk outcomes or remodeling phenotypes. A third approach of ML is reinforcement learning. This method would lead the ML system to continuously interact with its environment to learn ideal decision strategies through a reward system [71]. Although reinforcement learning is still new in the context of cardiovascular applications, it carries great promise for evolving treatment strategies and real-time decision support. Each of these branches of ML holds promise and multiple strengths; however, they do not come without limitations. Supervised learning offers higher predictive accuracy when the usage of labeled data is available, while unsupervised learning excels identifying hidden relationships that suit discovery driven analyses; however, unsupervised learning approaches are quite difficult to validate in clinical settings [72,73]. Reinforcement learning is theoretically powerful, but can present limitations in terms of data demands, ethical oversight, and computational power [74]. Machine learning techniques involve a variety of different methods, including logistic regression, which still remains the cornerstone of risk modeling [75]. Logistic regression assumes that the relationship between predictors and outcome probability is linear [76]. This method provides a simple to follow and transparent approach to early prognostic research. However, a limitation of logistic regression is that, due to this simplistic approach, it tends to fall short when nonlinear data and nonlinearities interact, such as the biomechanical and hemodynamic environment that exists within the aorta [77].

These limitations tend to hinder the implementation of ML-based systems in practice; thus, nonlinear and ensemble-based methods have been popularized lately [78]. Support Vector Machines (SVM) are an example of these systems, which are used to identify hyperplanes that separate outcome classes in high-dimensional feature spaces, often overcoming the limitations of linear-based methods that tend to fail. The performance of SVMs may suffer in the presence of excess noise or overlapping data distributions [79]. Random Forest (RF) and Gradient Boosting algorithms, including modern variants such as XGBoost, LightGBM, and CatBoost, take a drastically different approach by combining many different decision trees into ensemble systems that balance these biases and variance [80]. By accumulating the predictions generated from multiple weaker models, these methods can gain strong generalizations even when datasets are quite complex and heterogeneous [80,81,82]. Although these systems shine when it comes to these nonlinear complicated datasets, the interpretability of the output generated tends to suffer and, thus, hyperparameters should be carefully chosen to avoid overfitting [83,84].

There are other ML-based systems that serve more specialized purposes. LASSO regression is an example, which introduces a penalty term that tends to limit the complexity of the model, alongside activating an automatic feature selection mechanism [85]. This is especially effective for smaller datasets with multiple correlated predictors. Partial Least Squares (PLS) regression tends to simplify complex data by finding a small number of commonalities between datasets that best explains their relationships to clinical outcomes [86]. K-nearest neighbors is another example, the main method of which is to capture intuitive neighbor relationships to classify incoming new samples based on known cases; however, it can struggle with scalability as datasets expand [87]. Artificial Neural Networks (ANNs), although simpler than deep architectures, are models that are inspired by the biological connectivity of neurons, which helps to capture the nonlinear relationships in input data. ANNs need larger datasets and careful regularization to maintain stability and functionality [88].

Statistical approaches are not the only way that AI has been used in acute aortic syndrome research, with both mechanistic and hybrid methods also considered as key methods due to their ability to blend data-driven and physics-based reasoning. Finite Element Modeling (FEM), for example, enables detailed biomechanical simulations to approximate deformation, rupture risk, and wall stress. When used in combination with ML, FEM-driven methods can be used in predictive frameworks, thus filling in the gap between computational biomechanics and clinical prognosis [89]. Traditional ML tends to do better with structured data, but is quite limited when it comes to raw, complex data such as imaging and waveform data. This gap was addressed by Deep Learning. DL algorithms use multilayered architectures to automatically learn hierarchical feature representations from raw data without exact feature engineering. This level of autonomy makes them much more powerful, but computationally expensive and less interpretable when compared to ML. When applied to real life cardiovascular images, DL can derive prognostic information from CT (computed tomography) scans, magnetic resonance imaging (MRI), and echocardiographic datasets. Prior to DL, these tasks would have required manual segmentation [90].

Within DL, Convolutional Neural Networks (CNNs) have been popularized lately due to their particularly impactful architectures. CNNs are efficient at analyzing medical images through examining multiple small sections and reusing filters across the whole image. Through multiple convolutional and pooling layers, CNNs construct hierarchical representations of data, starting with simplistic features such as the edges and textures, to more clinically applicable structures such as aortic wall, false lumen morphology, and wall deformation. This makes them good at analyzing advanced geometric structures [91]. These CNNs have advanced subsets, such as U-Net models, which have further enhanced this process of medical image segmentation by allowing skip connections to preserve spatial resolution across encoding and decoding connections. Models such as U-Net have become an integral part of the foundation for automated segmentation systems that are capable of differentiating aortic root, aneurysm and dissection flaps with comparable accuracy to manual annotation [92].

Although these more developed systems have exceptional accuracy, it is important to consider barriers that challenge these systems, such as data dependency, susceptibility to domain shift across different scanners, and limited interpretability of the generated results. Research conducted in this field tends to highlight the need for explainable AI and hybrid modeling, which includes integrating the predictive power of deep networks while maintaining the transparency of traditional methods. These approaches may yield systems that could not only predict risk but also reveal the underlying pathologies that may further disease progression. This would be a key step toward translation into clinical settings [93]. As AI advances, understanding its decision-making processes is becoming a necessary concern. Explainable AI-based models aim to make predictions in a transparent and digestible way. This addresses a concept known as the black box problem [94]. Amongst these models, SHAP tends to stand out for its exceptional framework and interpretability. SHAP models are quite unique, as they extend the Shapley value concept originally proposed by Lloyd Shapley, which is rooted in cooperative game theory. This concept aims to distribute rewards fairly amongst players; the SHAP model applies this concept to machine learning. In this context, each feature within the AI-based model is given a Shapley value which correlates to the amount of contribution the respective feature contributes to the generation of the model’s prediction [95]. Through this model, key features of fairness are achieved, which include local accuracy, missingness, and consistency. This ensures reliable attribution of importance and is considered a viable method for bridging the gap between surgical/physiological features in relation to outcomes, thus making it highly relevant in the setting of risk prognostication in acute aortic syndromes.

Another system that has been found to represent machine-based prediction results in an interpretable manner is nomograms. Nomograms are visual representations of statistical models that provide tailored risk assessment scores by combining multiple predictors into one tool. Each predictor is given a weighted score based on the contribution to the outcome generated, such that the cumulative score aligns with the probability of a clinical event occurring. By condensing complex, multivariable models into a visual presentation, clinicians are able to utilize this system with ease as it is explainable and straightforward [96].

In conclusion, the ever-evolving field of AI to ML, and ultimately to DLs and CNNs, shows not only the capacity but the need to learn data from more complex datasets. When applied to this field, it moves from population-based methods to more tailored, image-driven prognostication. Each system contributes uniquely to the landscape of AI usage in this field. ML provides structure and interpretability, DL provides abstraction precision, CNNs deliver anatomical information, and Explainable AI models and nomograms provide transparency. When used together, they create opportunities to redefine how we predict, prevent, and understand outcomes for acute aortic syndromes.

## 3. AI-Based Prediction of Mortality in Acute Aortic Syndromes: From Early Proof-of-Concept to Clinically Oriented Models

Acute aortic syndromes (AAS), particularly aortic dissection (AD), are highly fatal conditions requiring urgent recognition and management. Mortality rates vary depending on the type of dissection, presence of complications, and treatment strategy. In Type A dissection, mortality can reach up to 50% without surgical intervention, whereas surgery markedly improves survival. Perioperative mortality has been reported at 17–25%, though more recent IRAD analyses indicate improved early surgical mortality of around 4–5% [97,98]. In Type B dissection, patients without high-risk features managed with optimal medical therapy (OMT) show in-hospital mortality of 10–15%. When complicated by rupture, malperfusion, or refractory hypertension, mortality rises to ≈50% under conservative management. Thoracic endovascular aortic repair (TEVAR) has become the preferred intervention in such cases, demonstrating ≈8% in-hospital mortality and reduced aorta-related deaths, although overall mortality showed no significant difference at 5-year follow-up. Several clinical risk models have been proposed to estimate operative mortality in acute type A aortic dissection (ATAAD), most notably the EuroSCORE II and the German Registry of Acute Aortic Dissection Type A (GERAADA) score. These tools have been shown to provide general prognostic insight; however, they remain limited in their ability to capture the full clinical complexity of aortic dissection. Both rely on static, preoperative variables and do not adequately account for management type, time to surgery, intraoperative parameters, hemodynamic instability, or evolving patient physiology—all of which substantially influence outcomes [99]. Given that acute aortic dissection affects approximately 3–6 individuals per 100,000 annually worldwide, a significant amount of clinical and procedural data has now accumulated [100]. This expanding data landscape provides a foundation for transitioning from conventional, population-based risk assessment to precision medicine approaches supported by advanced AI-driven tools, capable of integrating multidimensional clinical and imaging features to improve individualized mortality prediction.

Early attempts to apply artificial intelligence in aortic dissection date back to 2009 and 2010, when Macrina et al. [15] investigated the potential of artificial neural networks (ANNs) for mortality prediction after acute Type A repair. In 2009, ANNs showed promising performance for 30-day mortality, achieving an AUC of 0.90. The following year, the same group extended the approach to long-term survival, evaluating ANNs and support-vector machines (SVMs), with ANNs outperforming SVMs (AUC 0.87 vs. 0.83) [21]. Despite their time—when no standardized AI guidelines or reporting frameworks existed—the authors performed external validation of their model. It is important to note that the small sample size posed a potential risk of overfitting and limited real-time applicability. However, this pioneering effort should be praised as the first proof-of-concept study in this field, highlighting that capturing nonlinear interactions among clinical variables could substantially enhance mortality prediction in aortic dissection.

After more than a decade with limited progress, the use of artificial intelligence in aortic dissection resurfaced in the early 2020s, particularly for risk stratification and triage purposes. Tan et al. [14] developed a fusion decision model based on boosting trees—an ensemble of LightGBM, XGBoost, and CatBoost—to predict in-hospital or 30-day postoperative mortality after surgery for aortic dissection. Conducted during the COVID-19 pandemic, when surgical capacity and resources were restricted, this study demonstrated that AI could assist clinical teams in prioritizing high-risk patients and optimizing surgical timing under constrained conditions. The model achieved perfect prediction accuracy (100%) on the test set, while the individual base models reached accuracies of 0.8667 for LightGBM and 0.9833 for both XGBoost and CatBoost. Although AUC, sensitivity, and specificity were not reported, the analysis identified red blood cell transfusion, cardiopulmonary bypass time, and rectal temperature as the strongest predictors of early postoperative mortality.

Building on the concept of early risk assessment, Wang et al. applied a binary logistic regression model to emergency department (ED) data from patients presenting with acute aortic syndromes to predict in-hospital mortality before confirmatory imaging or surgery. Using six readily available clinical variables—digestive symptoms, pulse deficit, creatinine, lesion extension to the iliac vessels, pericardial effusion, and Stanford type A—the model achieved strong discrimination, with an AUC of 0.838 in the development cohort and 0.821 in an external validation cohort [17]. Together, these studies illustrate the growing clinical integration of AI as a practical decision-support tool, particularly for rapid triage and resource prioritization in the management of aortic dissection.

As the application of AI in aortic dissection evolved, researchers began developing intervention-specific models to refine postoperative risk assessment. Naazie et al. [16] developed the first clinically relevant TEVAR Mortality Risk Calculator to predict 30-day mortality following thoracic endovascular aortic repair (TEVAR) for intact descending thoracic aortic aneurysms (DTAA). Using data from 2141 patients across multiple institutions in the Vascular Quality Initiative (VQI) registry, the authors built a multivariable logistic regression model incorporating six independent predictors: age ≥ 75 years, coronary artery disease, American Society of Anesthesiologists (ASA) class IV/V, procedure urgency, prior carotid revascularization, and proximal landing zone < 3. The model achieved an AUC of 0.75, with internal cross-validation yielding a bias-corrected AUC of 0.73. Although the study was limited by a lack of external validation, it provided a robust foundation for procedure-specific risk stratification, supporting preoperative planning and shared decision-making in TEVAR candidates.

In a related effort, Zhang et al. [28] applied an enhanced AI framework (PSO-ELM-FLXGBoost) to predict 30-day mortality following total aortic arch replacement with frozen elephant trunk implantation in acute Type A aortic dissection. The model achieved an AUC of 0.8687, significantly outperforming conventional approaches. Using SHAP analysis, the study highlighted the impact of age, cardiopulmonary bypass time, D-dimer, ALT, and eGFR on perioperative mortality, emphasizing how advanced and interpretable AI systems can improve precision in postoperative outcome prediction (see Table 2 for details).

Collectively, these studies reflect a shift from general mortality estimation to procedure-specific, interpretable models, paving the way for precision risk assessment and tailored perioperative management in patients undergoing complex endovascular or open aortic repair.

While AI-based tools have shown promising potential for mortality prediction in aortic dissection, their clinical adoption depends on demonstrating clear advantages over conventional risk scores such as EuroSCORE II or the German Aortic Score. Without clear improvement in predictive accuracy or clinical usefulness, the extra complexity, time, and resources required for AI implementation may not be justified.

Liu et al. addressed this need for comparison through two large multicenter studies; both designed with robust datasets that included enough patient events to reduce the risk of overfitting and ensure reliable model performance. The first study [22], published in 2022, introduced the 5A score—an inflammation-based model for predicting operative mortality in acute Type A aortic dissection. Using data from 5014 patients across 13 centers, divided into derivation, internal validation, and external validation cohorts, the authors applied an XGBoost algorithm including 12 variables such as the systemic thrombo-inflammatory (STI) index. The model achieved excellent discrimination with AUCs of 0.873, 0.875, and 0.845 in the three cohorts, outperforming the GERAADA score (AUC 0.709).

The second study [29], published in 2024, developed the Bio-XGBoost model, focusing on inflammatory, coagulation, and metabolic biomarkers from 3110 surgically treated Type A dissection patients. It achieved AUCs of 0.943 in the derivation and 0.884 in external validation cohorts, again outperforming EuroSCORE II and GERAADA. Beyond its predictive accuracy, the study also showed how risk stratification could guide treatment decisions—patients classified as middle-to-high risk benefited from anti-inflammatory therapy with ulinastatin, resulting in shorter mechanical ventilation times.

These two studies strongly suggest that adding biological markers to AI models can greatly improve the prediction of mortality in aortic dissection. However, since both studies were conducted in Chinese populations, further testing in Western countries is needed to avoid population bias and to create models that can be used reliably worldwide.

Following the development of these high-performing short-term prognostic models, recent efforts have expanded toward predicting outcomes beyond the acute and perioperative phases. Although early mortality remains a critical endpoint in aortic dissection, understanding long-term survival is equally essential, as it supports individualized follow-up and informs key management decisions such as whether to pursue surgical repair or continue optimal medical therapy. Addressing this broader clinical perspective, Zhang et al. [27] and Cai et al. [26] focused on extending AI-based prediction to 1-year and multi-year outcomes, respectively.

Zhang et al. [27] developed a Treebag ensemble model that achieved an AUC of 0.91 for predicting 1-year mortality in patients with Type A dissection. SHAP analysis identified surgical intervention, β-blocker use, and higher systolic blood pressure as key protective factors—some of which are modifiable—highlighting the influence of treatment strategies on post-discharge outcomes. Expanding this horizon, Cai et al. [26] applied a Support Vector Machine (SVM) model to predict long-term survival after surgical repair of Type A dissection, incorporating intraoperative and postoperative variables such as cardiopulmonary bypass time, aortic cross-clamp duration, and plasma transfusion volume. Their model achieved AUCs of 0.91, 0.85, and 0.88 in the training, internal, and external validation cohorts, respectively. SHAP analysis confirmed that longer operation duration, renal dysfunction, and elevated inflammatory markers were dominant predictors of late mortality—providing clinically actionable insights for surgical planning and long-term monitoring.

These studies are valuable not only for extending AI’s role in mortality prediction but also for integrating explainable AI approaches, providing interpretable insights that strengthen clinical confidence in machine learning–based decision tools.

Liu et al. [20] focused on frailty assessment by developing an automated U-Net–based algorithm to measure lean psoas muscle area (LPMA) from CT scans in 428 patients with complicated Type B aortic dissection (TBAD) undergoing TEVAR. Using a threshold of 395 cm^2^·HU, low LPMA was linked to significantly higher 30-day (4.6% vs. 1.0%) and long-term (21.6% vs. 5.2%) mortality and remained an independent predictor of all-cause mortality (HR 5.62, *p* < 0.001). This study introduced an automated, frailty-based biomarker, highlighting that muscle quality and physiological reserve meaningfully affect outcomes beyond conventional clinical variables.

Overall, AI applications in aortic dissection have evolved from early proof-of-concept models to clinically oriented, interpretable tools addressing both short- and long-term outcomes. Recent studies emphasize the importance of integrating clinical, laboratory, and imaging data to enhance mortality prediction and support individualized management. However, as with many AI models in the broader cardiovascular literature, most studies continue to face challenges such as small sample sizes, lack of external validation, and potential overfitting [101,102]. These limitations will be discussed in detail in a later section of this review, where potential strategies to address them will also be proposed.

## 4. AI-Based Prediction of Composite Endpoints After Aortic Dissection Surgery

Mortality remains the most severe and measurable outcome, but it does not fully represent the clinical impact of aortic dissection surgery. Patients often survive the acute phase but face a high risk of serious postoperative complications that profoundly influence long-term prognosis and quality of life. These include neurological injury, renal failure requiring dialysis, respiratory failure, sepsis, and multiorgan dysfunction, among others. Focusing solely on mortality also poses methodological challenges, as its relative rarity in contemporary surgical cohorts can limit sample size and reduce statistical power for model development. Expanding the predictive scope toward major morbidity and composite adverse outcomes provides a more comprehensive understanding of patient trajectories and offers greater clinical utility. Consequently, several recent studies have applied artificial intelligence (AI) and machine learning (ML) to predict these high-impact complications following aortic dissection surgery.

Aortic dissection surgery remains one of the most complex and high-risk cardiovascular procedures. Operations such as Total Arch Replacement, Frozen Elephant Trunk (TAR + FET), Hemiarch Replacement, and Thoracic Endovascular Aortic Repair (TEVAR) are associated with substantial postoperative morbidity. Recognizing this, recent AI-driven studies have aimed not only to predict mortality but to anticipate the broader spectrum of adverse outcomes that define patient recovery.

Carroll et al. [59] provided one of the earliest examples by analyzing 602 patients undergoing hemiarch replacement using the Extreme Gradient Boosting (XGBoost) algorithm. Their model aimed to predict severe postoperative complications—stroke, death, or new renal replacement therapy—and achieved 88% accuracy with an AUC-ROC of 0.76. SHAP analysis highlighted key predictors such as low hemoglobin, older age, need for aortic root replacement, intraoperative red blood cell transfusion, and renal function. This study illustrated how AI can detect subtle clinical interactions and improve early recognition of patients at elevated risk.

Building on this foundation, Xie et al. [58] applied a similar XGBoost framework to 380 patients undergoing surgery for acute type A aortic dissection. Their model focused on postoperative adverse outcomes (PAOs), defined as severe complications including renal failure, respiratory failure, neurological damage, sepsis, and low cardiac output syndrome. The algorithm achieved an AUROC of 0.761 and an accuracy of 69.3%, identifying preoperative aortic regurgitation, operation duration, inflammatory markers, and circulatory arrest time as key determinants. Both Carroll and Xie demonstrated that AI could uncover nonlinear patterns between perioperative variables that are often missed by conventional regression, offering a clearer picture of morbidity risk.

The potential of AI further expanded with Lu et al. [60], who combined clinical and imaging data to predict adverse events after TEVAR in 369 patients with uncomplicated type B aortic dissection. Unlike earlier studies that relied solely on clinical predictors, Lu’s team integrated inflammatory and coagulation biomarkers—such as C-reactive protein and albumin—with deep learning–derived radiomic features describing aortic morphology, including false lumen volume, true lumen diameter, and intimal flap geometry. Their hybrid model achieved an outstanding AUROC of 0.985 and validation accuracy of 92%, significantly outperforming models based only on clinical or imaging data. By merging physiological and anatomical information, this study underscored the power of multimodal AI approaches to capture both the biological and structural determinants of aortic disease progression.

Luo et al. [61] then carried this work further through a comprehensive study of 635 patients undergoing Total Arch Replacement with Frozen Elephant Trunk (TAR + FET). They tested ten machine learning algorithms and nearly two hundred model combinations to predict major adverse outcomes (MAOs), including death, stroke, renal failure requiring dialysis, respiratory complications, and pleural injury. The best-performing model combined Random Survival Forest (RSF) and Gradient Boosting Machine (GBM) methods, achieving a C-index of 0.919 and an AUROC above 0.85. SHAP analysis identified coagulation markers (INR, APTT), bilirubin, hemoglobin, albumin, platelet count, neutrophil count, and ascending aortic diameter as key predictors. Patients at higher risk more frequently had preexisting renal or coronary disease, malperfusion, or prior thoracic surgery. Luo’s work demonstrated that ensemble AI methods can effectively integrate clinical, biochemical, and anatomical factors to predict complex postoperative trajectories.

When considered together, these studies reveal a clear evolution in AI applications for aortic dissection—from models predicting isolated endpoints like mortality to more comprehensive frameworks addressing the full spectrum of serious complications. Across different procedures and populations, XGBoost and ensemble models consistently achieved strong predictive performance, while SHAP-based interpretability helped bridge the gap between model complexity and clinical understanding. Although most of these studies remain limited by retrospective single-center designs and lack of external validation, their findings reinforce an important principle: the burden of aortic dissection surgery extends far beyond survival. By focusing on morbidity and composite adverse outcomes, AI-based models can provide richer, clinically actionable insights—allowing clinicians to identify vulnerable patients early, guide intraoperative decisions, and personalize follow-up care.

## 5. Role of AI in Predicting Acute Kidney Injury and Acute Renal Failure

Acute kidney injury (AKI) remains one of the most prevalent and life-threatening complications following surgical repair of acute aortic dissection (AAD), particularly in Stanford type A dissections. With incidence rates as high as 50–70% in this subgroup, the burden of AKI is both clinically and economically significant [103,104]. Several pathophysiological mechanisms have been implicated in its development, including hemodynamic instability, renal hypoperfusion, and a systemic inflammatory response related to cardiopulmonary bypass and surgical trauma [105,106]. Despite advances in surgical and perioperative care, AKI continues to be strongly associated with prolonged hospitalization, higher morbidity, and increased incidence of postoperative complications and in-hospital mortality [107].

Recent advancements in artificial intelligence (AI), particularly machine learning techniques, have facilitated the development of predictive models to assess AKI risk post-AAD. These approaches have gained increasing attention for their ability to synthesize complex clinical data and provide predictive insights, potentially allowing for earlier intervention and risk stratification. In our literature review, we identified seven studies that implemented machine learning algorithms to predict AKI in this high-risk patient population. The most commonly used machine learning models included Random Forest (RF), Extreme Gradient Boosting (XGBoost), LightGBM, Support Vector Machines (SVM), Artificial Neural Networks (ANN) and CatBoost. These models promise to enhance decision-making, identify high-risk patients earlier, and support precision medicine approaches in perioperative care.

Initial studies in this field primarily aimed to build and evaluate ML models to predict AKI using clinical, laboratory, and surgical parameters. Techniques such as logistic regression, random forest (RF), XGBoost, LightGBM, SVM, and ANN were commonly employed. One of the earliest large-scale efforts, conducted by Liu et al. [46] focused on predicting severe AKI (KDIGO stage III) following total aortic arch replacement (TAAR) for Stanford type A dissection. Their study combined multiple feature selection techniques such as LASSO, SVM-RFE, and RF. Ultimately, they found that the ANN significantly outperformed traditional logistic regression, achieving an AUC of 0.938. Similarly, Chen and Lu et al. [44] developed models for AKI prediction in a Chinese cohort undergoing type A AAD surgery, using LightGBM and applying SHAP explainability techniques to identify the most influential variables such as ICU ventilation time, postoperative hourly urine output, diuretic use, and heart rate. These studies marked a shift toward more data-rich, high-dimensional modeling, moving beyond basic preoperative labs to include intraoperative dynamics and ICU trends.

Despite differences in population and algorithm, certain predictors consistently emerged across models. Preoperative serum creatinine, blood urea nitrogen (BUN), intraoperative bleeding, postoperative lactate, red blood cell transfusion, and BMI were frequently reported as strong contributors. In several models, advanced biomarkers—such as cystatin C, myoglobin, CK-MB, and urinary N-acetyl-β-D-glucosaminidase (uNAG)—also provided substantial predictive value, especially in models targeting severe AKI or renal replacement therapy [44,45,46].

The study of Li Xinsai et al. [42] highlighted the heterogeneity within AAD populations by stratifying models according to Stanford type. They built separate models for type A and type B AAD and found that RF performed best for type A (AUC = 0.760) and LightGBM for type B (AUC = 0.734). While common predictors across both types included creatinine, BUN, uric acid, duration of mechanical ventilation and length of ICU stay, subtype-specific variables were also identified. For Stanford type A, important predictors included WBC count, platelet count, D-dimer, volume of plasma transfused, and cardiopulmonary bypass time. For Stanford type B AAD, NT-proBNP, potassium levels, APTT, systolic blood pressure, and the presence of intraoperative renal arteriography were unique features. This shift toward subtype-specific modeling reflects a broader movement in AI research, away from one-size-fits-all models toward more precise, population-tailored prediction tools.

Despite promising internal validation metrics, external validation remains rare. Most models report high AUCs during training or cross-validation, but their performance may degrade when applied to independent datasets. Two studies conducted robust external validation. In the first, Wei et al. [43] compared seven algorithms and found CatBoost to perform best (AUC = 0.876 in training, 0.712 in external validation) Similarly, Li J. et al. [40] developed a model for predicting postoperative acute renal failure and demonstrated stable performance using XGBoost (AUC = 0.82 in internal validation, 0.81 in external validation). These results underscore the importance of external testing to detect overfitting and ensure generalizability, which are key requirements for clinical implementation.

Moreover, the focus of studies is not restricted to acute kidney injury alone. Zhou et al. [41] built models to predict both AKI and paraplegia following thoracoabdominal aortic aneurysm repair. The results indicated that the Random Forest model was the most precise for predicting AKI, while the Linear SVM was superior for predicting paraplegia, both achieving an average AUC of 0.89. Meanwhile, other researchers focused specifically on more severe renal outcomes. Lie et al. [45] developed an XGBoost-based model to predict the need for continuous renal replacement therapy (CRRT), achieving an AUC of 0.96. Peak intraoperative lactate, transfusion volume, renal artery involvement, cystatin C, myoglobin, and CK-MB were among the most influential predictors. Such efforts highlight the flexibility of machine learning approaches in identifying both broad and high-risk clinical scenarios, thereby enabling early risk stratification and effective resource planning.

While AI-driven AKI prediction in AAD surgery holds significant promise, several limitations hinder its clinical translation. One major concern is the lack of external validation. Only a limited number of studies utilized independent cohorts, making most models susceptible to overfitting. Additionally, variability in AKI definitions poses a challenge. Although KDIGO criteria are commonly employed, inconsistencies in staging and timing reduce comparability across studies. Real-time applicability is another issue; clinical implementation remains challenging. Moreover, these models are often difficult to understand; although some use tools like SHAP or decision curve analysis to explain their results, many remain hard for clinicians to interpret, which limits their trust and use in practice. Future research should prioritize large, multicenter datasets, prospective validation, and real-time deployment. Incorporating explainable AI tools is crucial for clinical use.

AI-based models have emerged as powerful tools for predicting AKI following acute aortic dissection surgery. These systems offer a deeper understanding of risk dynamics and have demonstrated promising results across various algorithms and patient populations. With the growing interest in model explainability, external validation, and personalized modeling, it is suggested that AI will become an integral part of postoperative risk management in cardiovascular surgery.

## 6. AI-Driven Prediction of Thoracic Aortic Aneurysm Growth and Postoperative Complications

Thoracic Aortic Aneurysms (TAA) occur when there is dilation of the thoracic aorta due to an intrinsic weakness in the vessel wall. This condition is mostly asymptomatic; however, over time the diameter of the aorta increases and can eventually result in catastrophic consequences such as rupture, dissection, or intramural hematoma (IMH). Any of these conditions are potentially life-threatening and need immediate surgical correction. In the current landscape, the risk and treatment decisions of TAA is largely based on aneurysm diameter, with other factors considered including symptoms experienced and underlying conditions such as connective tissue disease. However despite this framework, AAS may still occur in aneurysms smaller than the threshold for surgical intervention, this necessitates the creation of methods to predict adverse outcomes of TAA accurately. With the introduction of AI/ML, many new discoveries are being made with regard to expanding the parameters surrounding TAA to predict patient outcomes more accurately. A study performed by Chiu et al. [37] analyzed the mechanical properties of over 400 aorta samples from 31 patients, both control and diseased. Due to a large number of potential predictive factors, machine learning technique, Least Absolute Shrinkage and Selection Operator (LASSO), was used to create a predictive model for aortic growth rate over time. They found that increasing aortic strain, indicating greater distensibility, is associated with growth over time, potentially identifying patients at risk for dissection or rupture. This may prove a useful metric in the surveillance and risk assessment of TAA. A two part study by Geronzi et al. [38,39] also investigated aneurysm growth rates. The first study [38] used a retrospective dataset of 50 patients with at least two 3D studies pre-operatively, using CT or MRI Angiography. Using ML classifiers, they determined four shape features to calculate aneurysm growth rate, Diameter, Diameter-centerline ratio, External-internal line ratio and Tortuosity. It was found that SVM, using all 4 features, had better specificity and sensitivity for accurately classifying aneurysm growth potential over diameter alone. The follow-up study [39] included 70 patients using the same inclusion criteria. With a more extensive dataset, statistical shape analysis was used to extract global shape features from principal component analysis and Partial Least Squares (PLS). Regression methods were then used to directly infer aortic aneurysm growth rate from the computed shape features. The PLS regression model achieved an impressive Root Mean Square Error (RMSE) of 0.066 mm/month. These findings demonstrate that diameter alone is no longer the most accurate way to account for TAA risk. ML is paving the way for advancements in clinical medicine by discovering possible improvements to current guidelines, allowing for more factors to be taken into account for more personalized and effective patient care plans. Applications of ML are not only confined to pre-operative circumstances either, two studies by [47,48] investigated remodeling after Thoracic Endovascular Aortic Repair (TEVAR). The first study [48] retrospectively analyzed 503 patients with Stanford Type B Aortic Dissection (TBAD) who underwent proximal TEVAR, 38 clinical and imaging variables were collected. 4 ML algorithms, namely LR, ANN, RF and SVM, were compared in their ability to predict distal aortic enlargement of > 5 mm and distal aortic aneurysm formation. The LR model performed best in predicting distal aortic enlargement with a high sensitivity while the ANN model predicted distal aortic aneurysm formation with the highest specificity. The high sensitivity of LR can help identify high risk patients who need more close monitoring and follow-up. The high specificity of the ANN model could accurately identify patients with a low risk of distal aortic aneurysm and hence avoid over treatment. The second study [47] compared two conventional deep learning methods against one another in predicting negative aortic remodeling and reintervention after TEVAR. Negative aortic remodeling is the leading cause of late reintervention after proximal thoracic endovascular aortic repair (TEVAR) for Stanford type B aortic dissection (TBAD), and poses a great challenge to endovascular repair. Pre-operative CT Angiography data of 147 patients were used. The Point Cloud Neural Network (PC-NN) performed significantly better than the other models in predicting both negative aortic remodeling and reintervention. These results could allow clinicians to tailor management to reduce patient risks.

## 7. AI Applications for Predicting Rupture Risk and Location in Aortic Aneurysm and Dissection

Early computational approaches demonstrated that mechanical stress and wall tension, rather than size alone, determine rupture potential. Koru et al. [30] employed a hybrid FSI–AI framework using patient-specific aortic geometries derived from CT angiography. FSI simulations quantified deformation, wall shear stress, and the safety factor under pulsatile flow, confirming that rupture risk rose sharply with increasing aortic diameter—up to seven-fold higher at 48 mm compared to normal aortas. An Artificial Neural Network (ANN) trained on these simulation data predicted rupture risk with a near-perfect R^2^ = 0.986, providing a fast and accurate surrogate for time-intensive FSI analyses.

Similarly, Liang et al. [32] developed a machine-learning surrogate for finite element analysis (FEA) to predict rupture risk in ascending aortic aneurysms. Using a Statistical Shape Model (SSM) derived from 3D CT images of 25 patients, they generated 729 virtual aneurysm shapes and calculated the Pressure Risk Ratio (PRR) as a measure of rupture risk. The SVM model accurately classified high- and low-risk aneurysms (95.6% accuracy), while the SVR predicted continuous rupture risk with minimal error (0.0332), demonstrating that geometric shape features can effectively capture biomechanical vulnerability beyond traditional diameter-based criteria.

Transitioning from simulation-based frameworks to clinical datasets, Lin et al. [35] developed and compared logistic regression, random forest (RF), support vector machine (SVM), and convolutional neural network (CNN) models to predict early rupture (≤72 h post-CTA) in 200 patients with acute Type A aortic dissection (ATAAD). The CNN model achieved the highest performance (AUC 0.99, accuracy 0.90), followed by RF (AUC 0.94) and logistic regression (AUC 0.91). Independent predictors included older age, female sex, ventilator use, elevated lactate and WBC levels, and key imaging parameters such as maximum aortic diameter >48 mm and false lumen/true lumen area ratio >2.12.

In a larger retrospective cohort, Wu et al. [33] trained a random forest model on 1133 TAAD patients to predict in-hospital rupture. Sixteen predictors were identified, with periaortic hematoma emerging as the strongest risk factor. The model achieved an AUC of 0.994 in training and 0.752 in external validation, highlighting both the power and the overfitting risks inherent in high-dimensional clinical models.

Similarly, Dong et al. [34] analyzed CTA imaging features from 564 ATAAD patients to identify predictors of preoperative rupture. The false lumen/true lumen diameter ratio ≥4:1 and false lumen diameter ≥36 mm were the most influential parameters; both strongly associated with rupture before surgery. This work emphasized the geometric and hemodynamic importance of false lumen expansion, particularly in DeBakey II dissections, where pressure buildup is confined to the ascending aorta.

Together, these studies reveal a gradual evolution from purely mechanical models to clinically interpretable, imaging-driven AI tools capable of quantifying rupture risk across multiple stages of aortic disease.

Beyond risk magnitude, recent research has also explored the spatial prediction of rupture location. He et al. [31] proposed a two-step ML framework using in vitro data from ascending thoracic aortic aneurysm (ATAA) specimens. The model first identified peak risk regions (PRR) where local tension buildup was greatest, then estimated local rupture strength to calculate a safety factor. LASSO regression accurately identified PRRs that matched true rupture sites in 13 of 15 specimens, and MLP regression predicted rupture strength with R^2^ = 0.905, suggesting that rupture location may be predictable from tension–strain responses within physiological pressure ranges.

In a complementary validation effort, Rourke et al. [36] directly compared predicted and observed rupture locations by overlaying pre- and post-rupture CT scans using iterative closest point registration. Finite element models successfully predicted rupture sites in patients whose scans were taken within two years before rupture, confirming that geometry-based strain modeling is time-sensitive and dependent on imaging quality.

Overall, these studies demonstrate that AI—whether used to replicate biomechanical simulations or to analyze clinical imaging—holds significant promise for improving rupture risk assessment and localization. However, most models remain early-stage and require larger, multicenter validation before integration into clinical decision-making frameworks.

## 8. Future Directions and Methodological Challenges of AI in Acute Aortic Syndromes

Despite the growing number of studies exploring artificial intelligence (AI) and prediction models in Acute Aortic Syndromes (AAS), the field remains in its early stages, with several limitations that hinder clinical translation. Most existing studies are based on small and single-center datasets, limiting generalizability and leading to model overfitting. The absence of external validation in many models raises concerns about reproducibility, while significant heterogeneity in data types and AI methodologies prevents any meaningful meta-analysis or holistic interpretation across studies. Such variation creates a wide and inconsistent data portfolio that complicates comparison and limits the ability to form unified conclusions. Although a meta-analytic synthesis may be theoretically possible, in practice, it remains unreliable due to these inconsistencies. Additionally, most models are static in nature, providing a single prediction rather than adapting dynamically to the patient’s evolving clinical or anatomical profile. Another key limitation is that AI-based prediction models are rarely compared directly with established clinical or surgical risk scores. Such comparisons are essential to determine whether AI truly outperforms conventional tools and addresses unmet needs in clinical decision-making.

To understand where the future of AI in AAS lies, it is first necessary to revisit the fundamental principles of how AI operates. Regardless of the model architecture, every AI system begins with data. The strength of any predictive model depends on the quality, quantity, and representativeness of its data foundation. Models trained on small or homogeneous cohorts are prone to overfitting, detecting coincidental rather than disease-related patterns. Therefore, the first step toward building reliable AI systems is ensuring an adequate sample size. The events-per-variable (EPV) ratio plays a key role in determining this balance, as highlighted by Riley et al. [108], who proposed a flexible calculation method adapted to the study design and modeling approach.

However, the availability of large datasets alone is insufficient. Equally important is the diversity of the data. Future research should encourage the inclusion of multi-center, multi-ethnic, and multi-modality cohorts to improve model generalizability and prevent bias. Once developed, models must also demonstrate robust external validation using independent datasets to confirm reproducibility and true predictive capability across institutions and populations. To support this, open-source data sharing and public availability of code should be actively encouraged, allowing independent researchers to replicate, test, and refine existing models. Such transparency will accelerate scientific progress, reduce duplication, and promote fair benchmarking across studies. Proper validation should assess not only discrimination (e.g., AUC, C-index) but also calibration—the agreement between predicted and observed risks. Calibration plots, calibration-in-the-large, and slope statistics are essential for evaluating whether a model systematically over- or underestimates risk. Without adequate calibration, even a model with excellent discrimination may fail in clinical use. These principles together form the foundation of data and model quality assurance.

Beyond data size and diversity, the content of the data is equally critical. The aorta is a dynamic and biologically active organ, continuously influenced by short-term factors such as blood pressure and strain, as well as long-term processes like inflammation and remodeling. Prediction models should therefore account for this temporal variability, integrating both acute and chronic determinants of disease progression. Moreover, outcomes in AAS are rarely determined by a single variable; they reflect complex systemic interactions. Thus, it is essential that all relevant clinical, hemodynamic, and biochemical contributors are incorporated into model development.

Future work should aim to develop integrated, multimodal models that combine these complementary data domains rather than relying on single-dimension predictors. Incorporation of radiomic signatures, biomechanical simulations, and hemodynamic indices may yield a more comprehensive understanding of disease behavior and rupture risk. However, as the number of predictors increases, so does the risk of overfitting. Hence, an optimal balance must be maintained between model complexity and sample size requirements. Internal validation using a separate dataset should be mandatory, ensuring that the model’s predictive performance is not inflated by overfitting to the training data. External validation, ideally performed on independent multi-center datasets, should then confirm the model’s generalizability across populations and clinical settings. Calibration assessment should accompany both internal and external validation to verify prediction reliability and clinical usability. In addition, direct comparison with conventional risk scores should be an integral step in future studies to clearly demonstrate whether AI-based models provide superior predictive power and clinical utility.

In the context of AAS, where disease progression is inherently dynamic, some studies have proposed dynamic nomogram models to capture temporal changes. However, these models were not truly adaptive, as they did not automatically update with new patient data over time. Instead, they mainly provided statistical relationships between variables rather than reflecting the ongoing physiological changes in individual patients. While this represents a valuable attempt to model the dynamic structure of the aorta, the next step should be the development of digital twins—patient-specific virtual simulations that can continuously integrate clinical, imaging, and hemodynamic data. Such models would allow real-time risk tracking and individualized treatment planning, marking a major step toward precision medicine in AAS.

Adherence to TRIPOD-AI reporting standards [109] and transparent methodology will be crucial to ensure reproducibility and clinical trust. Meanwhile, federated learning frameworks offer a practical solution to the challenges of data sharing and privacy, enabling multi-institutional collaboration without centralizing patient data. Ultimately, once AI systems achieve consistent performance, calibration, and explainability, the next step will be to conduct prospective, real-world validation studies to evaluate their clinical impact on decision-making and patient outcomes.

In the end, AI is not a magical solution. It is simply an advanced method of processing and interpreting complex data—another step in the long history of medical innovation. Like every major advancement in medicine, it should be approached with healthy skepticism but forward-looking optimism. When developed responsibly, AI has the potential not to replace clinical judgment but to enhance it, guiding medicine toward a more precise and personalized future.

## 9. Conclusions

Artificial intelligence has the potential to redefine how risk is assessed and managed in Acute Aortic Syndromes, moving the field toward a more precise, data-driven, and individualized approach to care. Yet, its promise can only be realized through scientific discipline—grounded in robust validation, methodological transparency, and fair comparison with established clinical risk scores. The next generation of research must go beyond proof of concept to demonstrate clinical relevance, reproducibility, and impact on patient outcomes. When guided by collaboration, ethical standards, and a commitment to clinical utility, AI will not replace human expertise but instead amplify it—transforming predictive modeling from theoretical potential into meaningful progress for patients with AAS.

## Abbreviations

Abbreviation	Full Term
AAS	Acute Aortic Syndromes
AKI	Acute Kidney Injury
ALT	Alanine Transaminase
ANN	Artificial Neural Network
APTT	Activated Partial Thromboplastin Time
ASA	American Society of Anesthesiologists
AUC	Area Under the Curve
AI	Artificial Intelligence
BMI	Body Mass Index
BNP	B-type Natriuretic Peptide
BUN	Blood Urea Nitrogen
CAD	Coronary Artery Disease
CatBoost	Categorical Boosting Algorithm
CK-MB	Creatine Kinase-MB
CNN	Convolutional Neural Network
CRRT	Continuous Renal Replacement Therapy
CT	Computed Tomography
CTA	Computed Tomography Angiography
CVRI	Cardiovascular Research Institute
DCR	Diameter-Centerline Ratio
DL	Deep Learning
eGFR	Estimated Glomerular Filtration Rate
EuroSCORE II	European System for Cardiac Operative Risk Evaluation II
FDA	Food and Drug Administration
FEM	Finite Element Modeling
GERAADA	German Registry of Acute Aortic Dissection Type A
HR	Hazard Ratio
ICU	Intensive Care Unit
IMH	Intramural Hematoma
INR	International Normalized Ratio
LASSO	Least Absolute Shrinkage and Selection Operator
LightGBM	Light Gradient Boosting Machine
LPMA	Lean Psoas Muscle Area
ML	Machine Learning
MODS	Multiorgan Dysfunction Syndrome
MRI	Magnetic Resonance Imaging
MVT	Mechanical Ventilation Time
NT-proBNP	N-terminal pro B-type Natriuretic Peptide
OMT	Optimal Medical Therapy
PC-NN	Point Cloud Neural Network
PCMI	Precision Cardiovascular Medicine & Innovation Institute
PLS	Partial Least Squares
PRR	Peak Risk Regions/Pressure Risk Ratio
RAM	Random Access Memory
RF	Random Forest
RMSE	Root Mean Square Error
R^2^	Coefficient of Determination
SHAP	SHapley Additive exPlanations
SVM	Support Vector Machine
SVM-RFE	Support Vector Machine Recursive Feature Elimination
SVR	Support Vector Regression
TAAR	Total Aortic Arch Replacement
TAA	Thoracic Aortic Aneurysm
TAR + FET	Total Arch Replacement with Frozen Elephant Trunk
TBAD	Type-B Aortic Dissection
TEVAR	Thoracic Endovascular Aortic Repair
TRIPOD-AI	Transparent Reporting of a Multivariable Prediction Model for Individual Prognosis or Diagnosis—Artificial Intelligence Extension
uNAG	Urinary N-acetyl-β-D-glucosaminidase
WBC	White Blood Cell
